# Supplementary material for: Complex picture for likelihood of ENSO-driven flood hazard
Source: Nat Commun. 2017 Mar 15;8:14796. doi: 10.1038/ncomms14796 (PMC5355947; doi:10.1038/ncomms14796)
Supplement: Supplementary Information — Supplementary Table, Supplementary Figures and Supplementary References [file ncomms14796-s1.pdf]

**Supplementary Table 1 | El Niño and La Niña years identified in the ERA-20CM SST data from 1901 to 2010.**			
**ERA-20CM El Niño Years**		**ERA-20CM La Niña Years**	
1902	1965	1903	1955
1904	1968	1906	1956
1905	1972	1907	1961
1911	1982	1908	1962
1913	1986	1909	1964
1914	1987	1910	1970
1918	1991	1916	1971
1923	1994	1917	1973
1925	1997	1924	1974
1930	2002	1933	1975
1939	2004	1938	1984
1940	2006	1942	1988
1941	2009	1943	1995
1951		1945	1998
1952		1949	1999
1957		1950	2007
1963		1954	

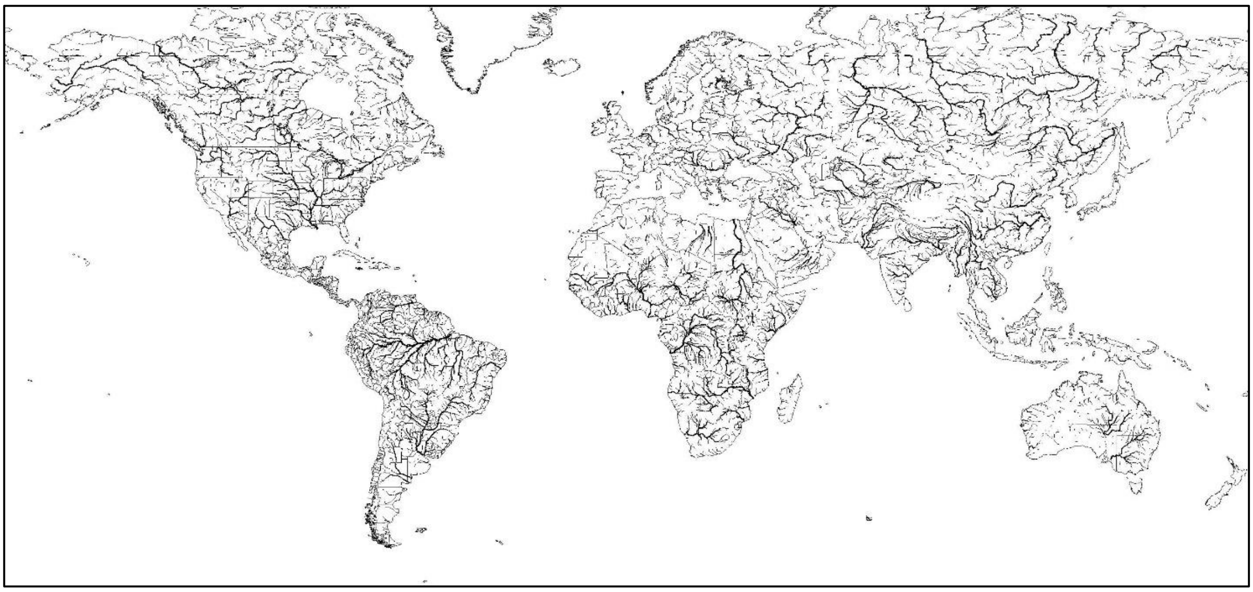

Supplementary Figure 1 | The CaMaFlood[1] 0.5^{o} global river network used in this study.

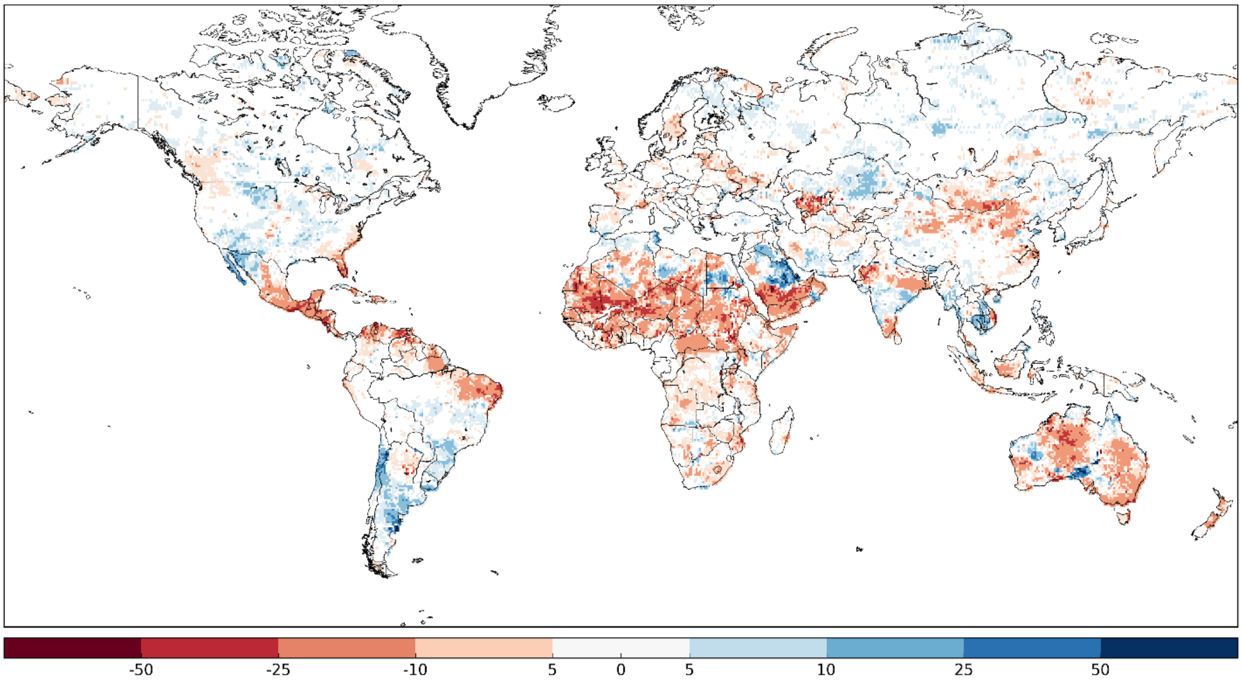

Supplementary Figure 2 | Percentage anomaly in the 100-year flood return period during El Niño. This replicates the analysis of Ward et al.[2] in order to ensure accurate estimation of the historical probabilities of ENSO-driven flood hazard using ERA-20CM-R.

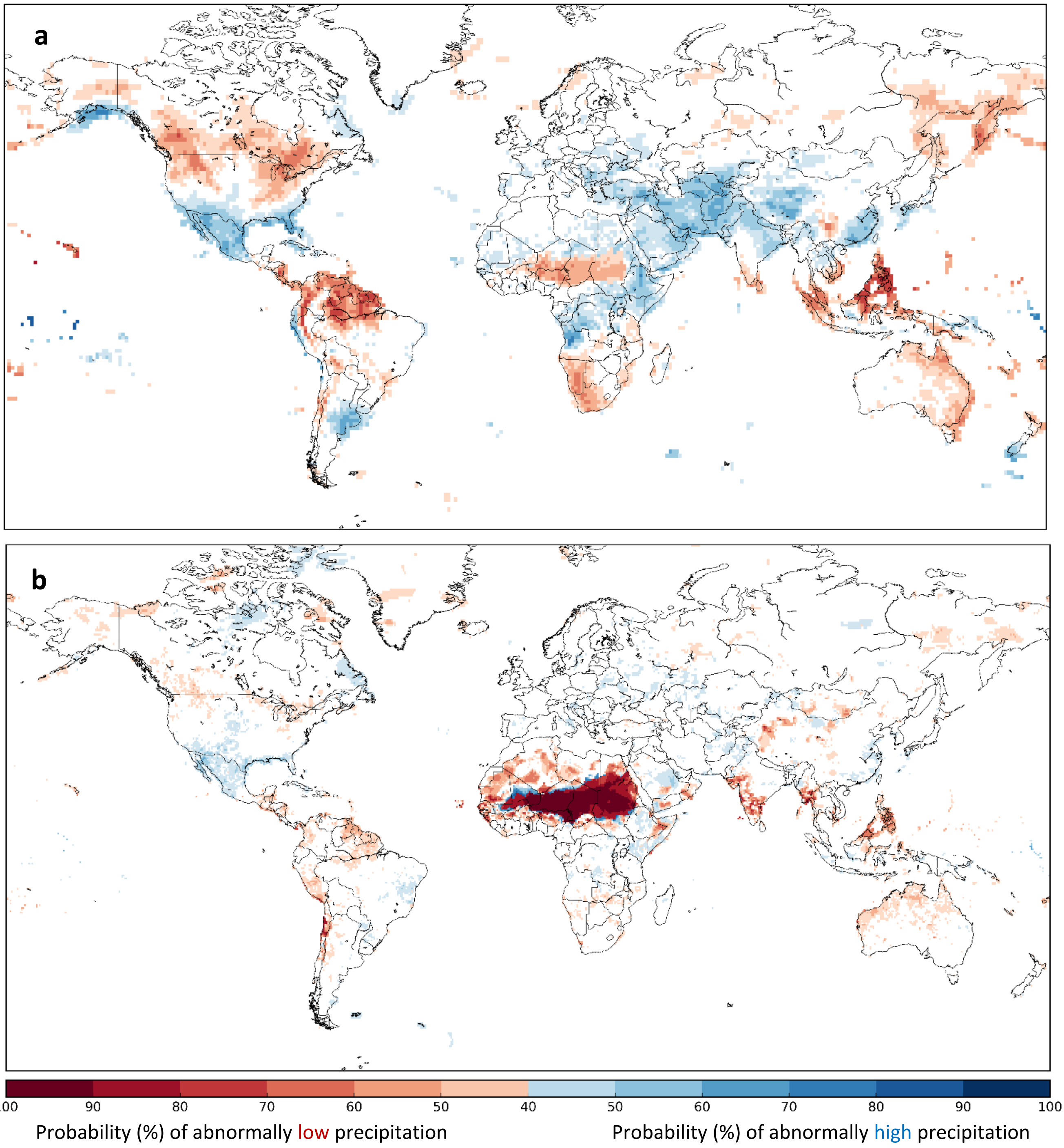

Supplementary Figure 3 | Probability of abnormally high (blue) or low (red) total monthly precipitation during the month of February during an El Niño. Based on total monthly precipitation exceeding the 75th percentile, or falling below the 25th percentile, of the 110-year (1901-2010) climatology. Using **(a)** the ERA-20CM dataset (based on the mean of the 10 ensemble members) and **(b)** the GPCC-FD[3] gridded precipitation dataset based on interpolated gauge observations. The large area of 100% probability (red) across northern Africa in (b) is most likely a result of the interpolation used to produce the GPCC-FD dataset in a region with few available observations.

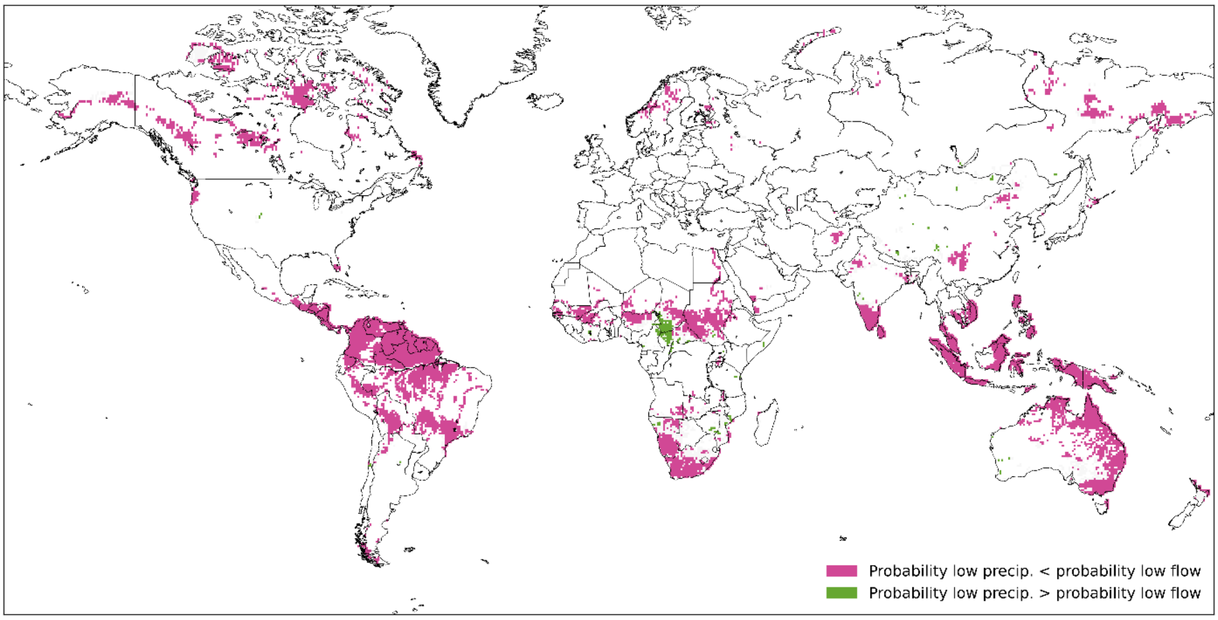

Supplementary Figure 4 | Comparison of historical probabilities based on precipitation and river flow. Regions where the difference in probability of abnormally low precipitation compared to probability of low river flow, in the month of February during an El Niño, is greater than 10% (based on the ensemble mean). Pink shading indicates that the probability of low precipitation is smaller than the probability of low river flow, while green shading indicates that probabilities are larger for precipitation.

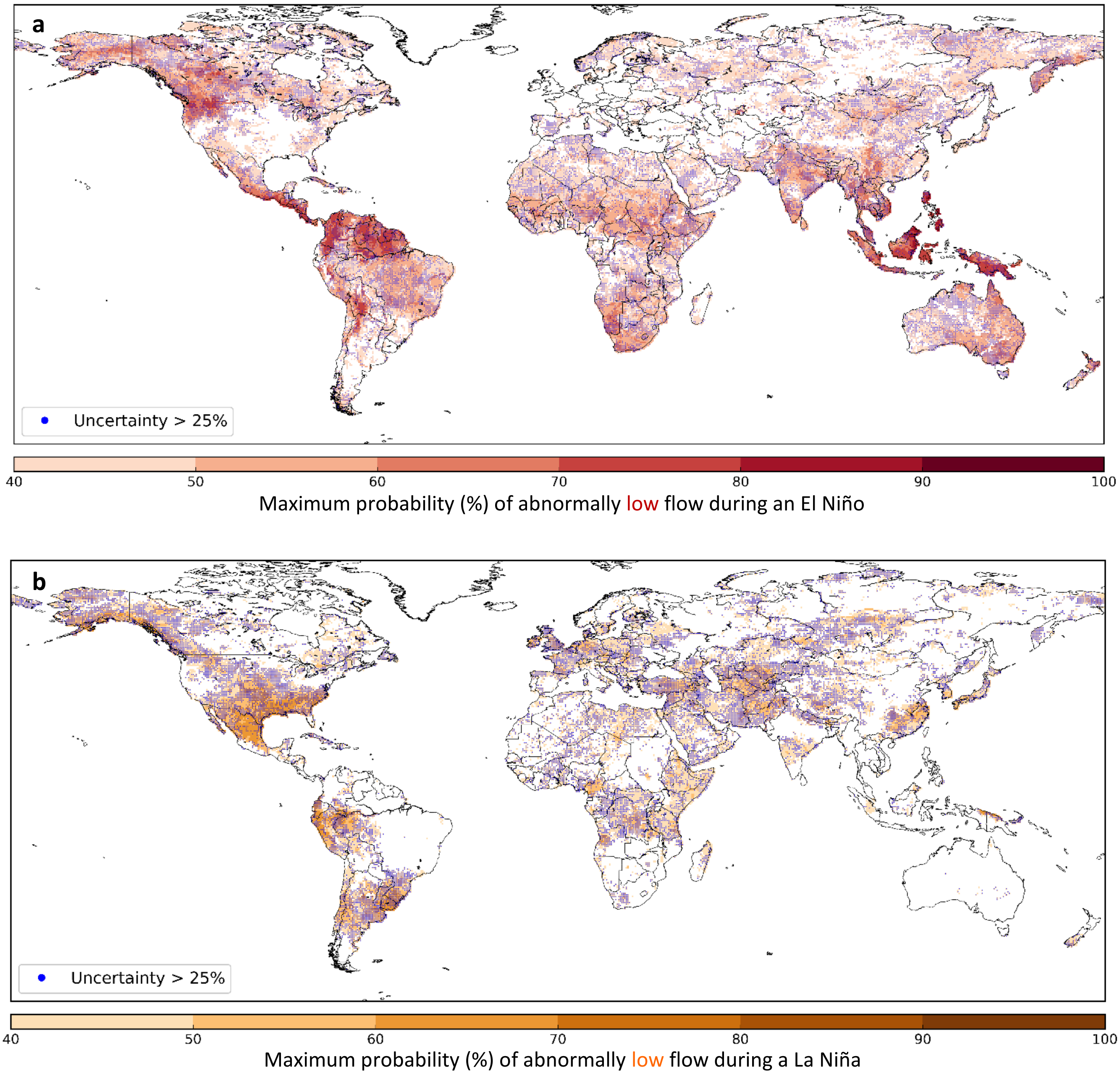

Supplementary Figure 5 | Maximum probability of abnormally low river flow in any month during (a) an El Niño event and (b) a La Niña event. Based on the mean of the 10 ERA-20CM-R ensemble members exceeding the 75th percentile, or falling below the 25th percentile, of the 110-year river discharge climatology during, or shortly after the decay of, an ENSO event. Stippling indicates where the uncertainty surrounding this probability is high, i.e. the range of the ensemble members exceeds 25% probability.

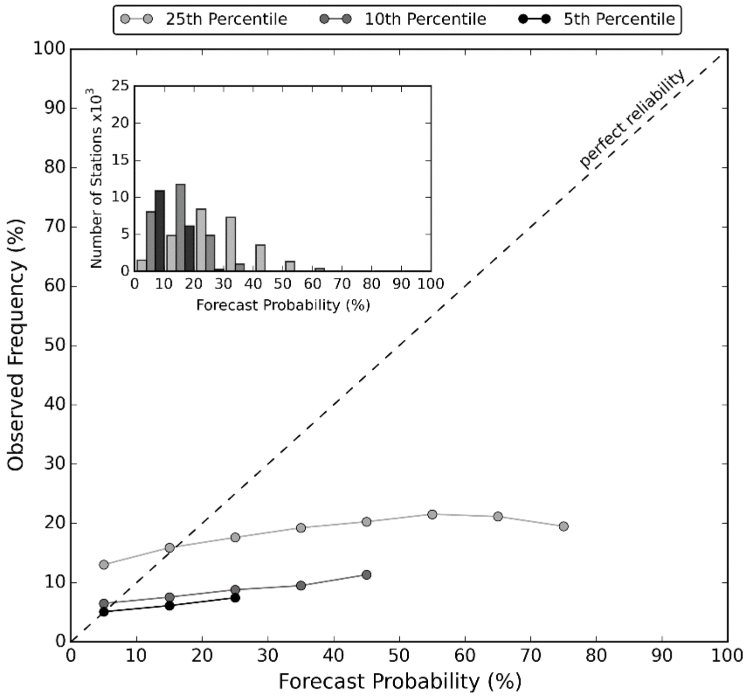

Supplementary Figure 6 | Reliability diagram comparing the forecast probability of abnormally high flow to the observed frequency in the GRDC observations. Results are included for exceedance of three river flow thresholds; the upper 25th, 10th and 5th percentiles. The results shown are an average across the 16 months from June during the El Niño year to the September following. Also shown is the number of available GRDC[4] observation stations in each percentage band.

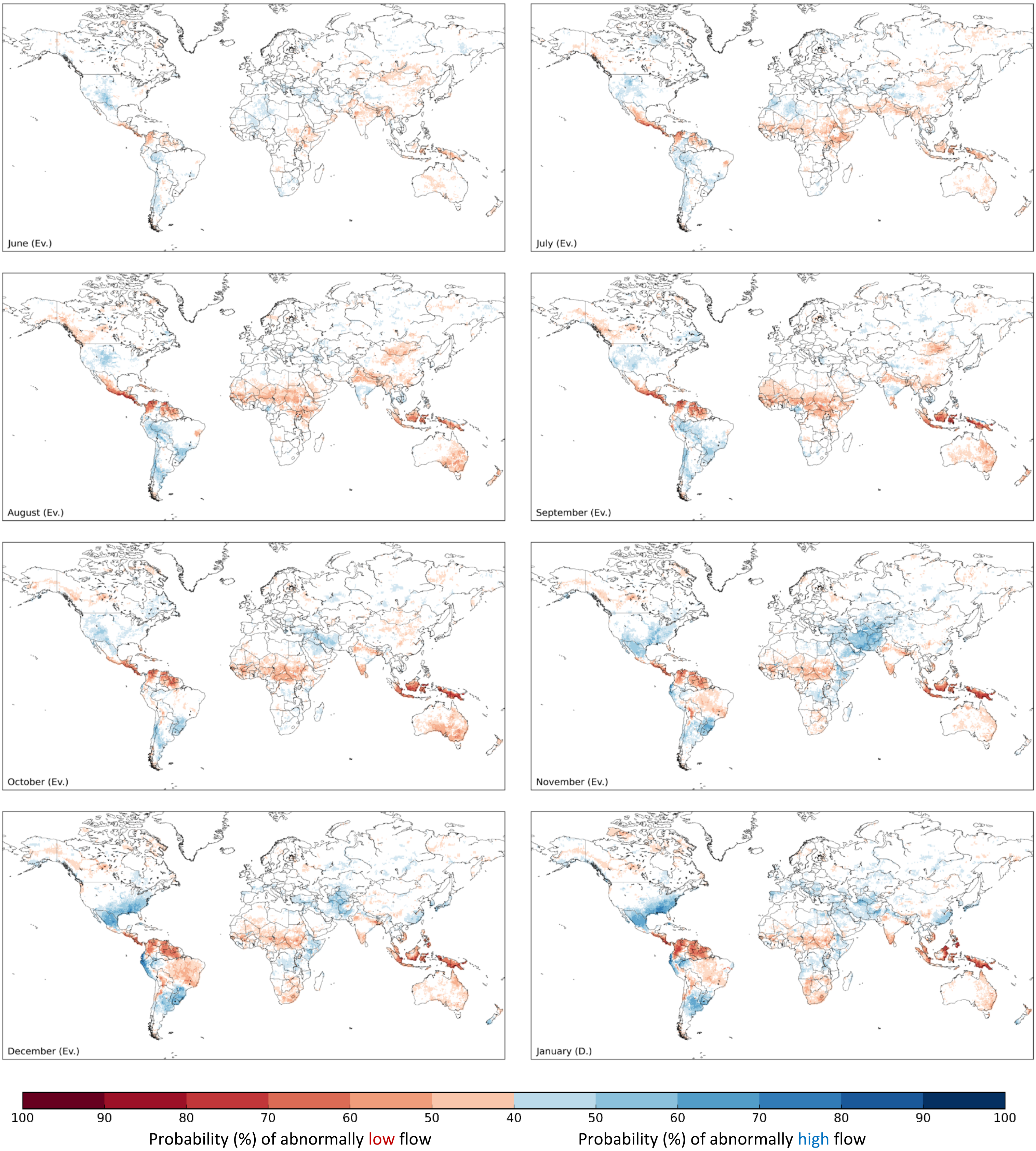

June (Ev.)
July (Ev.)
August (Ev.)
September (Ev.)
October (Ev.)
November (Ev.)
December (Ev.)
January (D.)
100 90 80 70 60 50 40 50 60 70 80 90 100
Probability (%) of abnormally low flow
Probability (%) of abnormally high flow

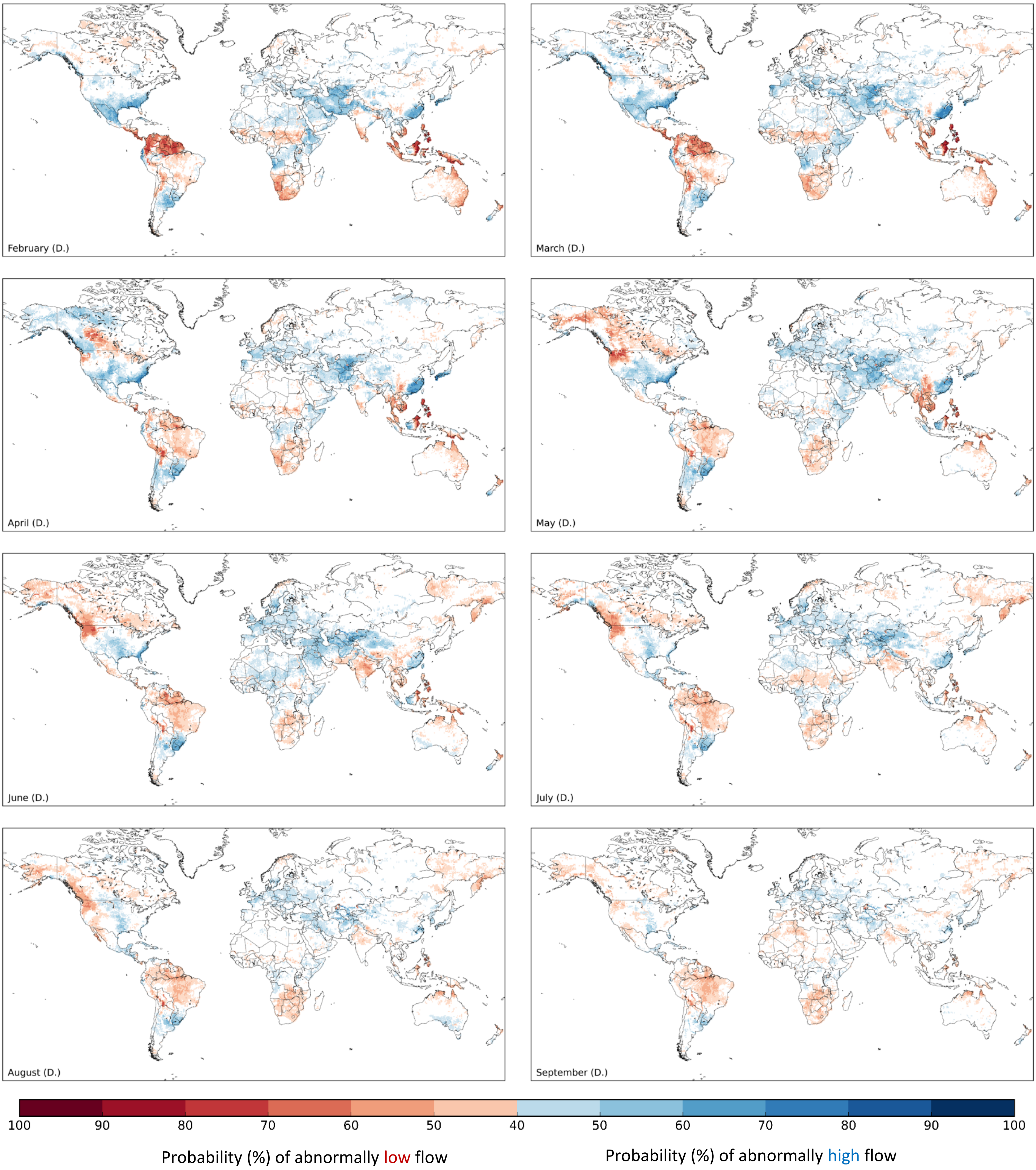

Supplementary Figure 7 | Historical probability of abnormally high (blue) or low (red) monthly mean river discharge during an El Niño. Each map shows the results for one month, based on the mean of the 10 ERA-20CM-R ensemble members exceeding the 75th percentile, or falling below the 25th percentile, of the 110-year ERA-20CM-R river discharge climatology. “Ev.” or “D.” indicates whether this map corresponds to the year in which the event typically evolves and peaks (“Ev.”), or the year in which the event is decaying (“D.”).

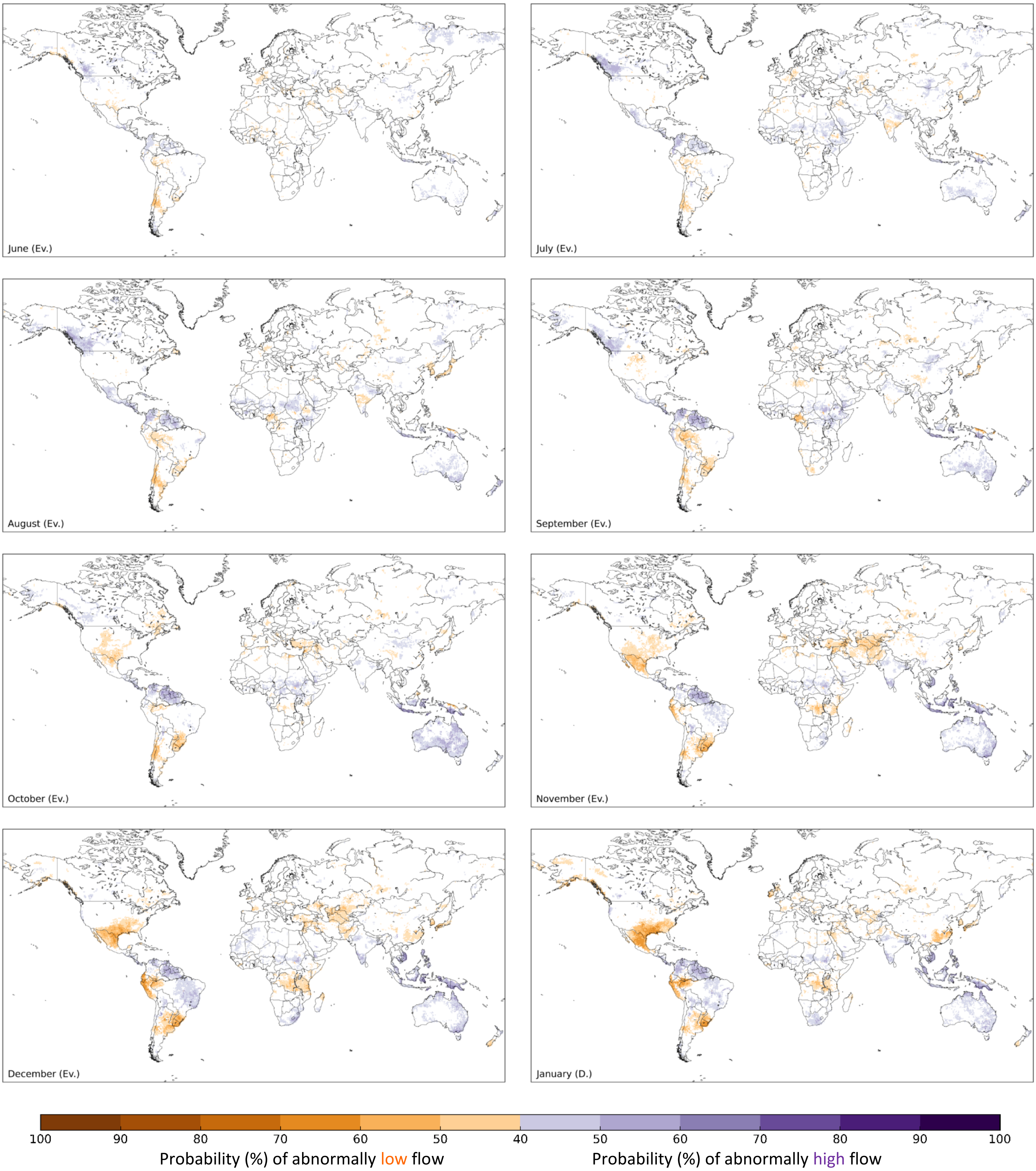
June (Ev.)
July (Ev.)
August (Ev.)
September (Ev.)
October (Ev.)
November (Ev.)
December (Ev.)
January (D.)
100 90 80 70 60 50 40 50 60 70 80 90 100
Probability (%) of abnormally low flow
Probability (%) of abnormally high flow

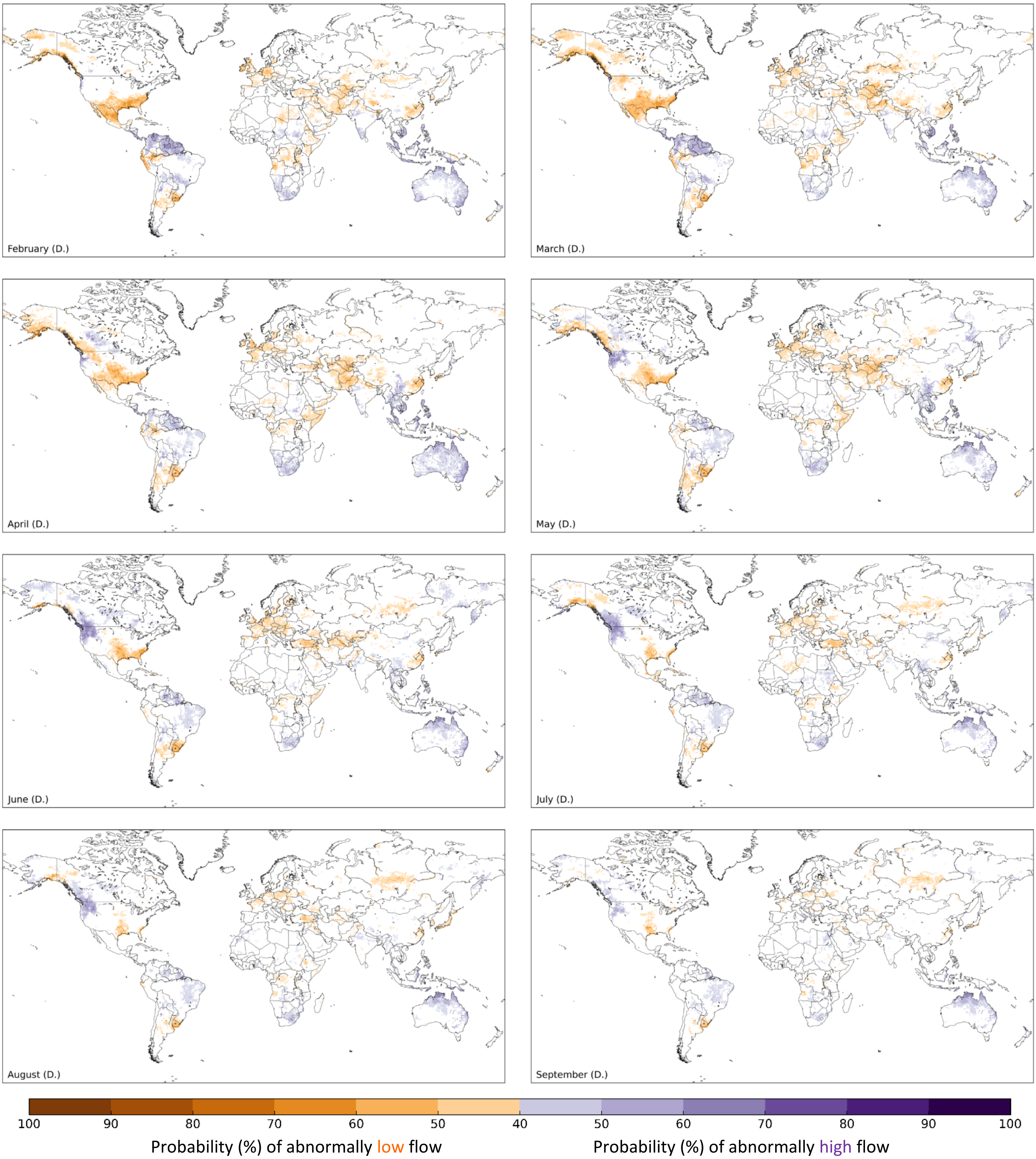

Supplementary Figure 8 | Historical probability of abnormally high (blue) or low (red) monthly mean river discharge during a La Niña. Each map shows the results for one month, based on the mean of the 10 ERA-20CM-R ensemble members exceeding the 75th percentile, or falling below the 25th percentile, of the 110-year ERA-20CM-R river discharge climatology. "Ev." or "D." indicates whether this map corresponds to the year in which the event typically evolves and peaks ("Ev."), or the year in which the event is decaying ("D.").

Supplementary References

1. Yamazaki, D., Kanae, S., Kim, H. & Oki, T., A physically-based description of floodplain inundation dynamics in a global river routing model. *Water Resources Research* **47**, W04501 (2011).

2. Ward, P. J. *et al.*, Strong influence of El Nino Southern Oscillation on flood risk around the world. *PNAS* **111** (44), 15659-15664 (2014).

3. Schneider, U. *et al.*, GPCC Full Data Reanalysis Version 7.0 at 0.5°: Monthly Land-Surface Precipitation from Rain-Gauges built on GTS-based and Historic Data (2015).

4. GRDC, River Discharge Data. *Global Runoff Data Centre. Koblenz, Federal Institute of Hydrology (BfG)* (2016).
